# In situ antigen modification-based target-redirected universal chimeric antigen receptor T (TRUE CAR-T) cell therapy in solid tumors

**DOI:** 10.1186/s13045-022-01246-y

**Published:** 2022-03-18

**Authors:** Zhichen Sun, Rutian Li, Yun Shen, Siyi Tan, Naiqing Ding, Ruihan Xu, Xinyue Wang, Jia Wei, Baorui Liu, Fanyan Meng

**Affiliations:** The Comprehensive Cancer Centre of Nanjing Drum Tower Hospital, The Affiliated Hospital of Nanjing University Medical School & Clinical Cancer Institute of Nanjing University, 210008 Nanjing, China

**Keywords:** Fusogenic nanoparticles, Antigen modification, Universal CAR-T cells, Cancer mmunotherapies, Solid tumors

## Abstract

**Background:**

Chimeric antigen receptor (CAR)-T cell therapy has demonstrated remarkable success in the treatment of hematologic malignancies, while the success has not yet been replicated in solid tumors. To some extent, the disappointing results can be attributed to the paucity and heterogeneity of target antigens in solid tumors since adequate antigens are the cornerstone for CAR-T cells to recognize and attack tumor cells.

**Methods:**

We established a target-redirected universal CAR-T (TRUE CAR-T) cell therapeutic modality, in which exogenous antigens are loaded onto fusogenic nanoparticles to achieve in situ modification of cell membrane in solid tumors, providing targets for subsequent CAR-T cell therapy. The modification effect was evaluated by flow cytometry and confocal microscopic imaging. The in vivo metabolism and biodistribution of fusogenic antigen loaded nanoparticles (F-AgNPs) was explored using near infrared living imaging. Then F-AgNPs mediated in situ antigen modification were cooperated with corresponding CAR-T cell therapy, and its antitumor efficacy was evaluated using immune function experiments and further investigated in different tumor models.

**Results:**

Using F-AgNPs, exogenous antigens were selectively modified onto tumor cell membranes through membrane fusion, spread deeper into tumor tissues through intercellular lipid transfer, further activating corresponding CAR-T cells and mediating antitumor immune responses towards multiple types of tumor cells, despite of their inherent antigen profiles. The cooperative treatment of F-AgNPs and CAR-T cell therapy successfully suppressed tumor proliferation and prolonged survival in both subcutaneous and peritoneally disseminated tumor models.

**Conclusion:**

The fusogenic nanoparticle-based in situ antigen modification overcome the limitation of target antigens paucity and heterogeneity in solid tumors, improving the efficacy and broadening the applications of CAR-T cells, thus establishing a novel TRUE CAR-T cell therapeutic modality with universal application and translational potential in immunotherapies for solid tumors.

**Supplementary Information:**

The online version contains supplementary material available at 10.1186/s13045-022-01246-y.

## Introduction

Recent developments in immunotherapy have been revolutionizing the treatment paradigms of malignancies. Among these immunotherapeutic strategies, T cells can be redirected to recognize certain tumor antigens and specifically kill targeted tumor cells through genetically engineered with chimeric antigen receptor (CAR), and the adoptive transfer of CAR-T cells has achieved impressive clinical efficacy in patients with hematological malignancies [1–3]. However, equivalent clinical benefits have not been demonstrated in patients with solid tumors yet, which collectively account for about 90% of cancer-related deaths [4]. Unlike hematological malignancies with uniformly expressed and relatively cancer-specific antigen targets, such as CD19, the antigen heterogeneity and high mutation diversities between cells in solid tumors make it scarcely possible to find universal tumor antigens that are stably expressed on multiple tumor types or even on different cells within a single patient’s tumor [5–7]. Existing CAR-T cell products could merely cover a minority of patients, even for those antigen-positive patients, only a subset of cells express corresponding antigens within tumor tissue, and the antitumor effect of CAR-T cells is commonly compromised due to insufficient expression of target antigens [8]. Moreover, high heterogeneity within solid tumors facilitates partial or complete loss of target antigens under therapeutic selective pressure, eventually leading to tumor outgrowth and escape from immunotherapies [9]. These factors severely limit the clinical application and therapeutic efficacy of CAR-T cell therapy in solid tumors [10].

**Fig. 1:**
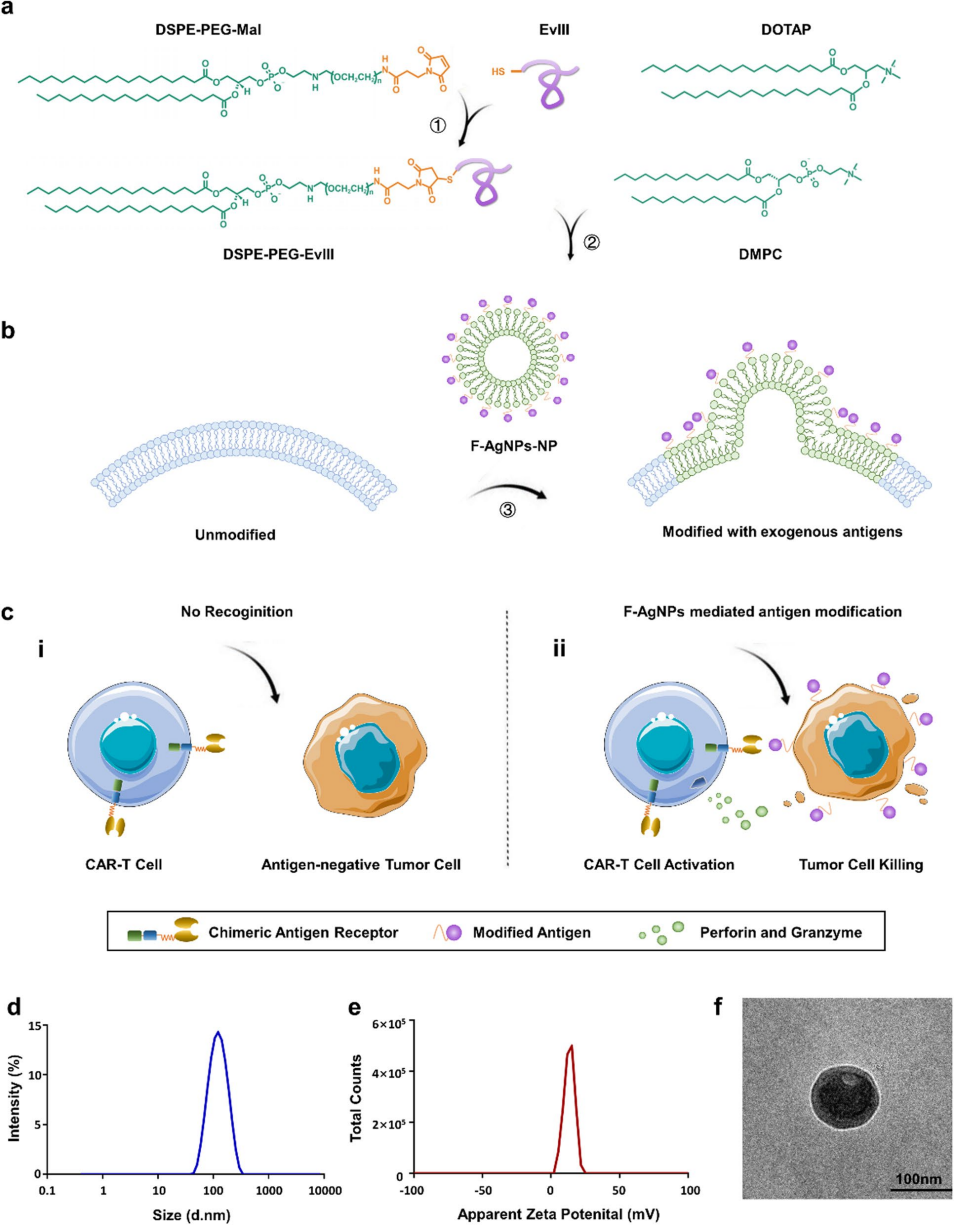
The construction of F-AgNPs and the proposed immunotherapeutic mechanism of TRUE- CAR-T therapy. **a** Schematic diagram of the synthesis of lipid-conjugated antigen peptides, DSPE-PEG-EvIII and the subsequent construction of F-AgNPs. **b** The schematic illustration demonstrating F-AgNPs modified exogenous antigens onto tumor cell membrane via membrane fusion. **c** In situ antigen modification of tumor cell membrane provides adequate targets for CAR-T cell recognition, enabling CAR-T cell to exert antitumor effects independent of tumor inherent antigen profiles (immunotherapeutic mechanism of TRUE- CAR-T cell therapy). **d** Particle size distribution of F-AgNPs. **e** Surface charge distribution of F-AgNPs. **f** The TEM image of F-AgNPs constructs, showing hollow near spherical structure of liposome. Imaged using JEOL 1200 EX TEM. Negative staining by 2% phosphotungstic acid. Scale bar represents 100 nm

Previous countermeasures towards the lack of antigen targets for CAR-T cell therapy mainly include multi-targets CAR-T cells which contain multiple CAR constructs in a single CAR-T cell product [11–13], and adapter-based universal CAR- T cells which can target different antigens through switching bispecific adapter with various specificity [14–16]. These pioneering studies have demonstrated some potential to improve the efficacy of CAR-T cells in solid tumors. Nevertheless, they are still restricted to tumors with suitable inherent antigens, which serve as the cornerstone for CAR T cells function, without which tumors could not be effectively recognized and destroyed. Herein, we proposed that if appropriate exogenous antigens can be in situ modified on solid tumors with favorable efficacy, stability, flexibility and safety, to provide adequate targets “redirecting” immune recognition, it would greatly broaden the application and improve the efficacy of CAR-T cell therapy. Fusogenic nanosystems were found as a good candidate [17]. The interaction pattern of plasma membrane fusion circumvents endocytosis pathway, efficiently loads cargo onto cytomembrane and achieves membrane specific modifications of living cells [18, 19]. Therefore, we sought to leverage the fusogenic nanosystem as an in situ antigen modification platform to overcome the paucity and heterogeneity of adequate target antigens which are pervasive in solid tumors, establishing a tumor inherent antigen-independent target-redirected universal CAR-T cell (TRUE CAR-T) therapy with practical potential in solid tumors (Fig. 1a–c).

## Methods

### Materials

EvIII peptide (LEEKKGNYVVTDHGSC, MW = 1778.4) and EvIII peptide with FAM conjugated at the N-terminus (FAM-EvIII) were purchased from GenScript Company (Nanjing, China). 1,2-Dimyristoyl-sn-glycero-3-phosphocholine (DMPC) and 1,2-dioleoyl- 3-trimethylammonium-propane (DOTAP) were purchased from (Avanti Polar Lipids, Alabaster, AL, USA). 1,2-Distearoyl-sn-Glycero-3-Phosphoethananolamine-N-[Maleimide (polyethylene-Glycol)-3400] (DSPE-PEG-Mal) was purchased from (Laysan Bio, Inc, USA).

### Cell lines

Human gastric adenocarcinoma cell line MKN45, MGC803, HGC27, human gastric mucosal epithelial cell line GES-1, human umbilical vein endothelial cells HUVECs, human peritoneal mesothelial cells HMrSV5 and mouse fibroblasts NIH-3T3 were purchased from the Cell Bank of Shanghai Institute of Biochemistry and Cell Biology. MKN45, MGC803, HGC27 and GES-1 were cultured in RPMI 1640 medium, HMrSV5 and mouse fibroblasts NIH-3T3 were cultured in DMEM medium. The media were supplemented with fetal calf serum (10%), penicillin (100 U mL^−1^) and streptomycin (100 μg mL^−1^). Human umbilical vein endothelial cells (HUVECs) were cultured in EBM-2 medium (Lonza, USA). All cells were incubated at 37 °C and 5% CO_2_, authenticated by checking morphology by microscopy. Cells were tested for Mycoplasma and only Mycoplasma free cells were used.

### Synthesis of DSPE-PEG-EvIII

DSPE-PEG-Mal was mixed with EvIII or FAM- EvIII at a 1:1 molar ratio in HEPES buffer (pH = 6.5). This reaction mixture was gently stirred at 4 °C for 24 h under nitrogen gas. After that, the resulting reaction mixture was placed in a dialysis bag (molecular weight cutoff = 2000 Da) and dialyzed in deionized water for 48 h to remove the free EvIII. The final solution in the dialysis bag was lyophilized and analyzed by matrix-assisted laser desorption/ionization-time-of-flight-mass spectrometry (MALDI-TOF MS).

### Nanoparticle preparation and characterization

DMPC, DSPE-PEG-EvIII and DOTAP were used to prepare nanoparticles. The molar ratios of DMPC, DSPE-PEG-EvIII and DOTAP used for F-AgNPs, P-NPs and NF-NPs were 72.5:4:23.5, 96:4:0 and 76.5:0:23.5, respectively. For DiI/DiR labeled F-AgNPs, the molar ratio of DMPC, DSPE-PEG, DOTAP and DiI/DiR was 72.5:4:23.5:1; For nanoparticles preparation, lipids were dissolved in chloroform and then completely dried by rotary evaporation. The remaining lipid film was hydrated using phosphate-buffered saline and then extruded through 100 nm membrane pores (Whatman, Little Chalfont, UK). The hydrodynamic size and zeta potential of nanoparticles were measured using dynamic light scattering (Zetasizer Nano ZS90; Malvern Instruments, Malvern, UK). The structural morphology was visualized by transmission electron microscope (JEOL 1200EX). Samples (20 μl) were prepared by dropping of nanoparticle suspension with a pipet gun and dropped onto the copper grid with carbon film for 3–5 min, absorbing the excess liquid by filter paper, and dropping 2% phosphotungstic acid for negative staining. Particle physiostability was observed by storing the formulations in PBS (or plasma) at 4 °C, and measuring the hydrodynamic diameter and surface charge every three days for a total of 3 weeks. The experiment was replicated three times and averaged.

### Antigen modification with F-AgNPs

MKN45 and MGC803 cells were seeded at the concentration of 1 × 10^6^ ml^−1^, (FAM-) F-AgNPs was added into cell culture medium at the indicated concentrations and incubated for 45 min in 37 °C at 5% CO_2_ incubator to achieve the modification of antigen peptide EvIII on the cell membrane. The cells were then washed with PBS and centrifuged (180*g*, 5 min, RT) to remove free reagents. If incubated with F-AgNPs without FAM fluorescence, tumor cells were successively incubated with mAb against EGFR vIII (L8A4, Absolute Antibody) and a secondary PE goat anti-mouse IgG antibody (Biolegend). The efficiency of tumor antigen modification with F-AgNPs was analyzed by flow cytometry. Cell vitality was assessed by CCK8 assay. To investigate the stability of antigen modification on cell surface, MGC803 cells were treated with mitomycin to inhibit mitosis, EvIII-FAM was modified onto tumor cells at saturation quantity as mentioned above. FAM-F-AgNPs-modified MGC803 cells were incubated in the complete medium in 37 °C at 5% CO_2_ incubator. At indicated time intervals, the cells were collected by centrifugation (180*g*, 5 min, RT) and analyzed by flow cytometry. All samples tested were suspended in flow cytometry staining (FACS) buffer and stained with the indicated antibodies for 30–60 min at 4 °C in the dark, washed twice, and resuspended in FACS buffer before analysis. Flow cytometry data were collected on Accuri C6 (BD Bioscience, NJ, USA) or CytoFLEX (Beckman Coulter, USA) and analyzed with FlowJo V.10.6.2 software.

### In vitro fluorescence cellular imaging

To observe whether EvIII peptide was successfully delivered to plasma membranes by F-AgNPs, we incubated MKN45 cells with F-AgNPs (containing 20 µg ml^−1^ DSPE-PEG-EvIII) for 45 min at 37 °C. After the thorough washing of free nanoparticles, cells were stained with DAPI and DiI to visualize nucleus or plasma membranes, respectively, and fixed with 4% paraformaldehyde. To observe tumor cell-selective antigen modification by F-AgNPs, we treated tumor cells (MKN45 and MGC803), normal human gastric mucosal epithelial cell line GES-1 and non-parenchymal cells (HUVECs, HMrSV5 and NIH-3T3) with F-AgNPs for 45 min. Cells were also stained with DAPI for 10 min at room temperature, and imaged using a confocal microscope (Nikon).

### Preparation of EvIII CAR-T cells

The blood collection procedure was carried out in accordance with the guidelines verified and approved by the Ethics Committee of Nanjing Drum Tower Hospital. All donors signed an informed consent for scientific research statement. Peripheral blood mononuclear cells (PBMCs) were isolated from samples from healthy volunteers by centrifugation on a Ficoll density gradient and suspended in AIM-V medium (Gibco, USA). PBMC were cultured in AIM-V medium for two hours to adherence. Non-adherent T lymphocytes were harvest and expanded in complete medium containing 90% AIM-V (Gibco, USA), 10% FBS serum (Gibco, USA), activated with 50 ng ml^−1^ OKT3 (eBioscience, USA), and 300 U ml^−1^ IL-2 (PeproTech, USA). After 48 h stimulation, OKT-3 activated T cells were transfected with the intended plasmids by Nucleofector 2B (Lonza, Germany). 10^7^ cells were washed with DPBS and resuspended in 100 μl transfection buffer (Amaxa Human T cells Nucleofector Kit, VPA-1002, Lonza, Germany). Program T-007 was selected. After transfection, cells were resuspended in pre-warmed AIM-V medium (500 μl) and cell culture medium was half replaced by fresh complete medium containing100 U ml^−1^ IL-2,10 ng ml^−1^ IL-7 (PeproTech, USA) and 10 ng ml^−1^ IL-15 (PeproTech, USA) every 2–3 days.

### T cell activation and cytotoxicity assays

MKN45 or MGC803 cells were modified with F-AgNPs at the indicated concentrations or control medium, and cocultured with EvIII CAR-T cells at an E:T ratio of 16:1. After incubation for 24 h at 37 °C, T cell activation was assessed by flow cytometry analysis of anti-CD69-APC (FN50, Biolegend), and anti-CD137-APC (M-A251, BD Bioscience). The supernatant fluids were harvested for cytokine quantification using the Human Th1/Th2 Cytokine Kit (BD Bioscience, NZ, USA). For cytotoxicity assays, MKN45 or MGC803 cells modified with F-AgNPs at different concentrations were incubated with EvIII CAR-T cells (E:T ratio = 10:1) for 5–6 h. The cytotoxicity was assessed by CFSE/PI labeling cytotoxicity assay.

### Experiments in multicellular tumor spheroids (MCTs)

The HGC27 cells (500 in 150 μl of complete media) were added to 96 Well Clear Round Bottom Ultra Low Attachment Microplate (Corning, USA) and allowed to grow up at 37 °C for about 72 h to attain the diameter approximately of 200 μm. MCTs were monitored with a microscope and the uniform and compact tumor spheroids were selected for the subsequent studies. To study the MCTs penetration of fusogenic nanoparticles, each type of nanoparticles was labeled with DiI. Established spheroids were incubated with F-AgNPs, NF-NPs or P-NPs (containing an equal amount of DiI) for 45 min at 37 °C, and washed to remove free nanoparticles. The spheroids were further incubated for 24 h to allow penetration of the cargo. After washing and fixing in 4% paraformaldehyde, tumor spheroids were scanned from the top to the middle with 10 μm intervals using confocal microscope (Leica, Germany) and the cross sections close to the center of the spheroids with similar diameters were then imaged at specific time points using confocal microscopy. The images were analyzed using ImageJ software. Cytotoxicity experiments in MCTs were conducted by exposing MCTs to control medium, F-AgNPs, EvIII CAR-T cells or F-AgNPs combined with EvIII CAR-T cells at the E: T ratios of 20:1 (F-AgNPs or control medium were added into MCTs, free nanoparticles were washed and removed after 45 min incubation, then EvIII CAR-T cells or control medium were introduced and co-cultured). After incubation for 12 h at 37 °C, the spheroids were washed, stained using a Viability/Cytotoxicity Kit (Biotium, California, USA), and fixed. Images were taken using a confocal microscope (Leica, Germany) as Z-stack projects scanning from the top to the middle of the MCTs with 10 µm intervals and then presented as maximum intensity projections. To quantification live/dead cells, the total cell area of each dye was measured using ImageJ.

### In vivo near-infrared fluorescence imaging

To investigate the biodistribution, metabolism and tumor targeting of F-AgNPs in tumor-bearing mice, F-AgNPs stained with near-infrared fluorescent probe DiR (Bridgen, China) were injected intraperitoneally (MGC803 peritoneal metastasis tumor model) or intravenously (MKN45 subcutaneous tumor model). At different time intervals, the mice were anesthetized and scanned using an IVIS Lumina III system (PerkinElmer, Massachusetts, USA). For resected tissue imaging, the mice were sacrificed under deep anesthesia. Tumors and main organs, including heart, liver, spleen, lung, kidney, and intestine, were excised and imaged.

### In vivo fluorescence tissue imaging

To observe antigen modification in tumor tissues, tumor models were generated by implanting 5 × 10^6^ MKN45 cells in 5-week old female nude mice. When the tumor volume reached about 100 mm^3^, mice were injected with either FAM-F-AgNPs or FAM-P-NPs (200 μl, 0.4 mM). Tumors were harvested at indicated time points and processed for immunostaining. Frozen sections were stained with CD31 using rabbit anti-mouse CD31 antibody (Abcam, UK) followed by the PE-conjugated goat anti-rabbit IgG (Abcam, UK) secondary antibody. After washing with PBS, the sections were mounted with DAPI (Beyotime, China) and analyzed using a confocal microscope (Leica, Germany).

### Xenogeneic mouse models

The Ethics Committee of Nanjing Drum Tower Hospital approved all experiments in this study. All animal procedures were carried out in compliance with guidelines set by the Animal Care Committee at Nanjing Drum Tower Hospital. Investigators were not blinded for animal studies. All efforts were made to minimize the number of animals used and their suffering. Mice were randomized on the basis of age and weight. For peritoneal metastasis tumor model, 5-week-old female BALB/c nude mice were injected intraperitoneally with 5 × 10^6^ MGC803 cells. For subcutaneous tumor model, 5-week-old female BALB/c nude mice were injected subcutaneously with 3 × 10^6^ MKN45 cells.

### In vivo antitumor efficacy

In the MGC803-luc mouse model, peritoneal tumor-bearing mice were randomized into four groups (*n* = 7). Nine days after tumor implantation (on day 9), F-AgNPs or PBS were given intraperitoneally, 4 h after F-AgNPs (200 μl, 0.4 mM) transfer, 1 × 10^7^ EvIII CAR-T cells were administered intraperitoneally. Treatment was repeated once 5 days after the start of treatment. Tumor burden was monitored before (day 8), 1 week (day 16), 2 weeks (day 23) and 3 weeks (day 30) after the start of treatment using the IVIS Lumina III system (PerkinElmer, Massachusetts, USA). Mice were weighed every three days. In the MKN45 model, subcutaneous tumor bearing mice were randomized in four groups (*n* = 7). Treatment was started when tumor volumes reached approximately 60 mm^3^. Mice were treated with intravenous injection of 100 ul PBS, F-AgNPs (100 μl, 0.8 mM), EvIII CAR-T cells (100 μl, 1 × 10^7^ EvIII CAR-T cells) and F-AgNPs + EvIII CAR-T cells, respectively. EvIII CAR-T cells were given 20 h after F-AgNPs transfer. The above treatment was given every five days for a total of twice. Tumor size was inspected every other day, calculated by the formula length × width^2^ × 0.5. For safety studies, one mouse from each group was randomly selected two weeks after the start of treatment, and main organs were collected for histology analysis. Organs were fixed in 10% neutral-buffered formalin, embedded in paraffin, sliced, and stained with hematoxylin–eosin (H&E).

### Statistical analysis

Graphpad Prism 8.0 (Graphpad software, San Diego, CA) and SPSS were used for all statistical analysis. Variance was similar between the groups that were compared statistically. No statistical methods were used to predetermine sample size. Data are presented as mean ± s.e.m. unless indicated otherwise. *p* < 0.05 was considered statistically significant.

## Results

### Construction of fusogenic antigen loading nanoparticles (F-AgNPs)

Epidermal growth factor receptor variant III (EGFR vIII) is a neoantigen derived from tumor-specific gene mutation, without expression on normal cells [20]. An antigen peptide from EGFR vIII (referred to here as EvIII), which can be effectively targeted by CAR-T cells, was chosen as a model antigen in this study, for its convenience for synthesis and modification [21]. The C-terminal cysteine residue of EvIII peptide provided the free sulfhydryl group to connect with the maleimide group of 2-distearoyl-sn-glycero-3-phospho-ethanolamine-N-maleimide (DSPE-PEG-Mal) through Michael addition reaction (Fig. 1a and Additional file 1: Fig. S1a) [22]. MALDI-TOF analysis showed the successful production of DSPE-PEG-EvIII (Additional file 1: Fig. S1b). F-AgNPs were constructed by controlling ratio of DSPE-PEG-EvIII, structural and cationic lipid components, using the film hydration method (Additional file 1: Fig. S1c) [23]. Dynamic light scattering (DLS) and transmission electron microscope data on the F-AgNPs confirmed a hollow near spherical construction, with an average hydrodynamic diameter of approximately 110 nm, and a cationic surface charge of approximately 10 mV (Fig. 1d–f and Table 1). The F-AgNPs formulations were physically stable in phosphate buffer solution for up to 21 days at 4 °C, and the particle size basically remained stable in blood plasma (Additional file 1: Fig. S2a–c).

**Table 1:** Lipid compositions and physical properties of synthetic nanoparticles used in this study

Nanoparticles^a^	Lipid composition (molar ratio, %)			Hydrodynamic size^b^ (nm)	Surface charge^c^ (mV)
	DMPC	DSPE-PEG-EvIII	DOTAP		
F-AgNPs	72.5	4	23.5	113.1 ± 1.24	11.2 ± 0.12
P-NPs	96	4	0	112.8 ± 1.18	− 9.2 ± 0.58
NF-NPs	76.5	0	23.5	144.2 ± 0.49	41.3 ± 1.4

^a^F-AgNPs, P-NPs, and NF-NPs denote fusogenic antigen loading nanoparticles, PEGylated nonfusogenic nanoparticles, and cationic nonfusogenic nanoparticles respectively

^b^Mean hydrodynamic sizes of the nanoparticles based on dynamic light scattering measurements (*n* = 3)

^c^Mean surface charges of the nanoparticles based on zeta-potential measurements (*n* = 3)

### F-AgNPs mediated cell membrane antigen modification and transfer

**Fig. 2:**
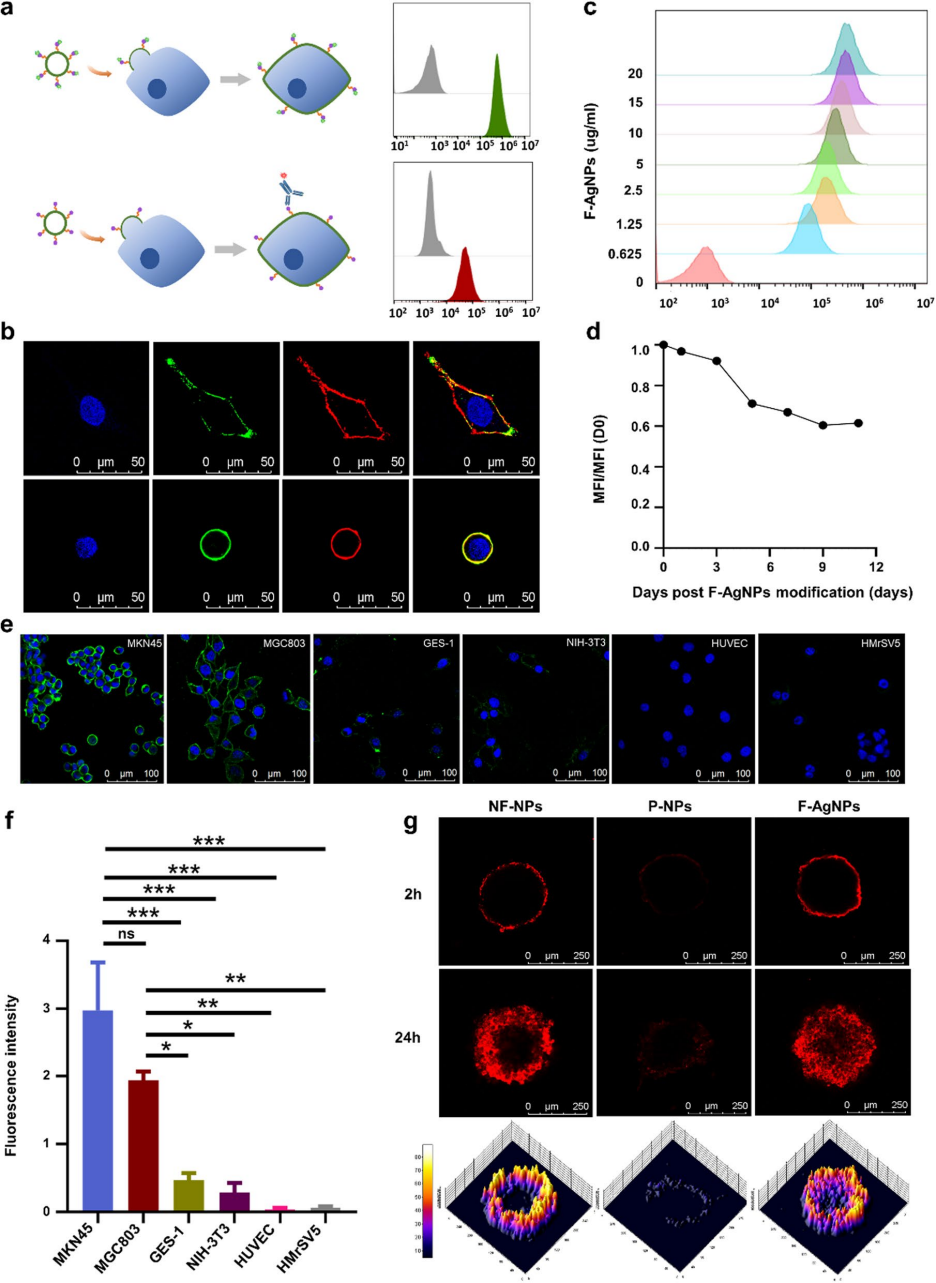
F-AgNPs mediated cell membrane antigen modification and transfer. **a** Schematic diagram of F-AgNPs mediated antigen modification onto tumor cells, and flow cytometry histograms of tumor cells without treatment (grey), incubated with FAM-F-AgNPs (green) or incubated with F-AgNPs followed by detection by PE-anti-EvIII mAb (red). **b** Localization of antigen peptides modified by F-AgNPs on tumor cells MGC803 (upper) and MKN45 (lower). Nucleus: DAPI (blue); antigen peptide: FAM (green); plasma membrane: DiI (red). Scale bar represents 50 μm. **c** Analysis of the dosage-effect relationship of F-AgNPs mediated antigen modification on tumor cells. **d** Flow cytometric analysis of stability of F-AgNPs mediated antigen modification over the culture periods. Data represent mean ± s.e.m., *n* = 3. **e**, **f** Representative images (**e**) and quantitative analysis (**f**) of F-AgNPs mediated antigen modification efficiency on gastric cancer cells, MKN45 and MGC803, and normal cells, GES-1, NIH-3T3, HUVEC and HMrV5 cells. Nucleus: DAPI (blue); antigen peptide: FAM (green). Scale bar represents 100 μm. Data are represented as mean ± s.e.m., *n* = 3. A one-way ANOVA was used for statistical analysis. ns not significant; *p < 0.05; **p < 0.01; ***p < 0.001. **g** Confocal images of HGC27 spheroids to assess penetration capacity of DiI delivered by NF-NPs, P-NPs or F-AgNPs. Scale bars, 250 μm

We first investigated whether F-AgNPs could efficiently modify tumor cells with antigen peptide by fusion with plasma membrane. Tumor cells were incubated with F-AgNPs-FAM or F-AgNPs for 45 min and washed thoroughly. The antigen peptide can be detected through FAM fluorescence or PE conjugated anti-EGFR vIII monoclonal antibody (Fig. 2a, Additional file 1: Fig. S3a, b, e, f).Confocal microscopy revealed that fluorescent peptide localized on plasma membranes rather than cytoplasm, indicating that F-AgNPs delivered antigen peptide throughout plasma membrane by direct membrane fusion, which were different from the endocytosis uptake pathway of conventional liposomes (Fig. 2b, Additional file 1: Fig. S3c, d). The density of cell surface antigen was controllable through adjusting the dose of F-AgNPs, with F-AgNPs containing 20 µg DSPE-PEG-EvIII created almost saturated modification of 10^6^ tumor cells without affecting cell viability, and the cell-surface antigen modification strategy suggested favorable stability (Fig. 2c, d and Additional file 1: Fig. S4). The modification selectivity was subsequently examined through co-incubating various cell lines with F-AgNPs-FAM to observe the antigen modification efficiency. After 45 min incubation, FAM-antigen peptides were effectively loaded onto the gastric cancer cells membranes, whereas the membrane-specific delivery of antigen was inefficient in normal gastric epithelial cells, fibroblasts, endothelial cells, and peritoneal mesothelial cells (Fig. 2e, f). Abnormal angiogenesis and high hydrostatic pressure are typical of solid tumor, which hindered the penetration of various therapeutic agents. Therefore, we chose multicellular tumor spheroids (MCTs) as in vitro model, mimicking the in vivo three dimensional cell masses, to study whether loaded antigen peptides could be transferred to neighbouring cell surfaces via intercellular lipid exchange. HGC27 tumor spheroids were treated with F-AgNPs, cationic nonfusogenic nanoparticles (NF-NPs) or PEGylated nonfusogenic nanoparticles (P-NPs) labeled with DiI for 45 min and washed thoroughly, incubated for another 24 h, and observed on 2 h and 24 h respectively (Table 1). Increasing migration of DiI from the periphery into the center was observed in the F-AgNPs-treated spheroids comparing with the NF-NPs- and P-NPs-treated spheroids, suggesting F-AgNPs formulation mediated superior penetrating capacity through multiple cell layers (Fig. 2g). These ex vivo studies initially confirmed our hypothesis that fusogenic nanoparticles could deliver antigens onto tumor cell membrane with a superior tumor penetrating capacity.

### F-AgNPs mediated antigen modification cooperates with CAR-T cell therapy in vitro

**Fig. 3:**
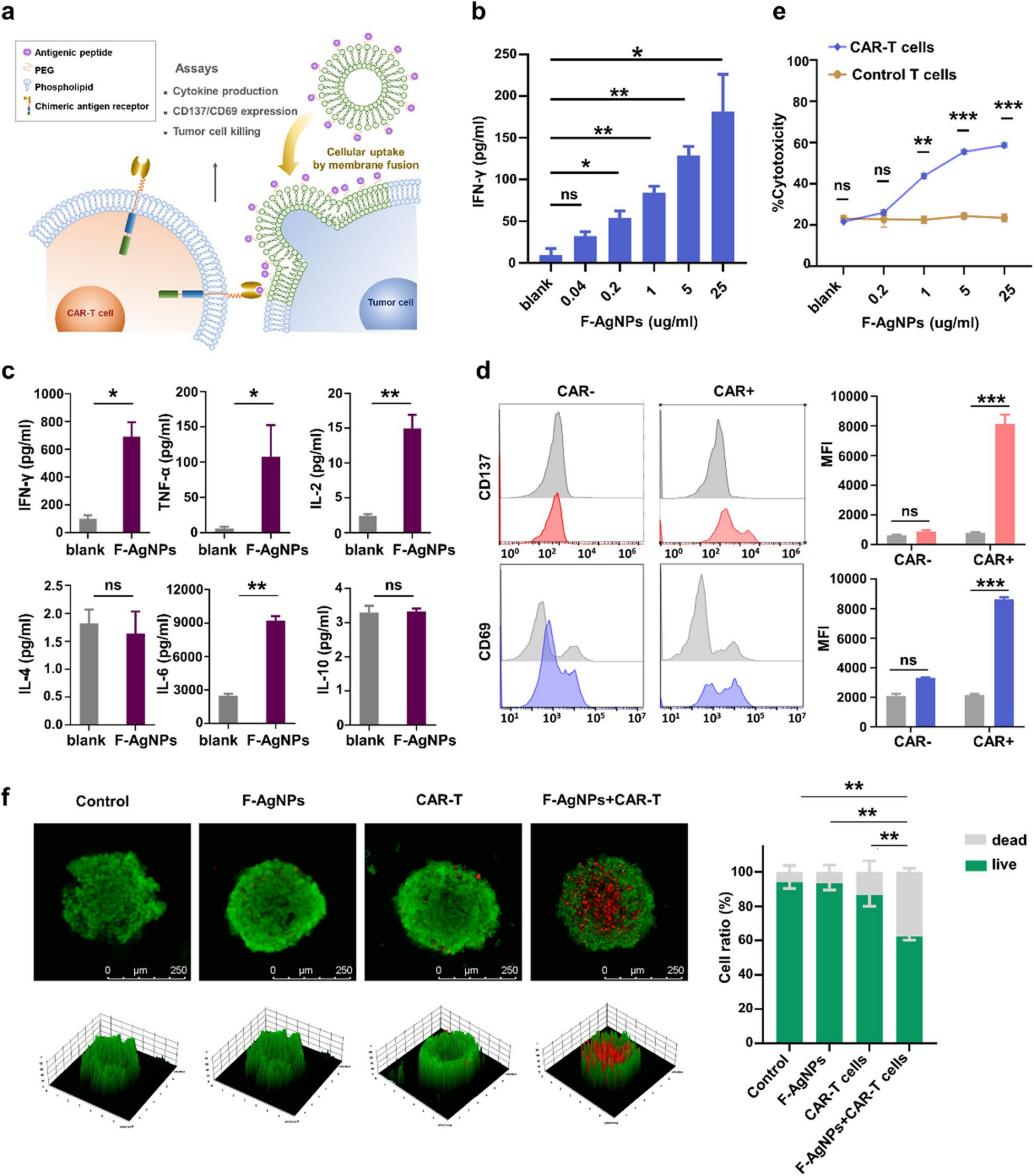
F-AgNPs mediated antigen modification cooperates with CAR-T cell therapy in vitro. **a** Schematic illustration of action mode of TRUE-CAR-T therapy and evaluation of in vitro antitumor response. **b** IFN-γ secretion of EvIII CAR-T cells after 24 h incubation with tumor cells (MKN45) treated by F-AgNPs of various concentration. Data are represented as mean ± s.e.m., *n* = 3. Student’s *t* test was used for statistical analysis. ns not significant; ns not significant; *p < 0.05; **p < 0.01. **c** Th1- and Th2-type cytokines secretion of EvIII CAR-T cells after 24 h incubation with F-AgNPs treated tumor cells. Data represent mean ± s.e.m., *n* = 4–5. Student’s *t* test was used for statistical analysis. ns not significant; *p < 0.05; **p < 0.01. **d** The expression of CD137 and CD69 on CAR negative- and positive-subpopulation after 24 h incubation with F-AgNPs treated tumor cells. Data represent mean ± s.e.m., *n* = 3–4. Student’s *t* test was used for statistical analysis. **e** Evaluating cytotoxicity of EvIII CAR-T cells and control T cells (mock transfection) towards F-AgNPs treated tumor cells (MKN45) and exploring the dose–effect relationships using CFSE/PI assay. E:T = 10:1. Data are represented as mean ± s.e.m., *n* = 3. Student’s *t* test was used for statistical analysis. ns not significant; **p < 0.01; ***p < 0.001. **f** Representative confocal images of HGC27 spheroids untreated, treated with F-AgNPs, CAR-T cells and F-AgNPs + CAR-T cells. E:T = 20:1 Dead cells:EthD-1 (red); live cells: calcein AM (green). Scale bar, 250 μm. Data represent mean ± s.e.m., *n* = 3. A one-way ANOVA was used for statistical analysis. *p < 0.05; **p < 0.01; ***p < 0.001

Given that target antigens could be effectively modified onto tumor cell membrane by F-AgNPs, whether the modified antigens could activate corresponding CAR-T cells was further investigated (Fig. 3a). EvIII specific CAR-T cells were cocultured with F-AgNPs treated tumor cells for 24 h, after which T lymphocyte activation markers and cytokine secretion were analyzed (Additional file 1: Fig. S5a-c). IFN-γ secretion assay revealed that F-AgNPs mediated engagement and activation of CAR-T cells in a dose dependent manner (Fig. 3b and Additional file 1: Fig. S6a). Subsequent analysis of multiple cytokines indicated that the F-AgNPs mediated CAR-T cell responses were characterized by the secretion of Th1-type cytokines, such as IFN-γ, TNF-α, and IL-2. Cytokines associated with Th2-type response, such as IL-4, IL-10 remained unchanged, expect for IL-6, which usually elevated in CAR-T cell therapy. The result is consisitent with the conventional cytokine secretion pattern of CAR-T cells (Fig. 3c). To further illuminate the antigen specificity of the F-AgNPs mediated antitumor response, the expression of activation markers on CAR negative- and positive-subpopulation were measured respectively. With F-AgNPs mediated antigen modification, CD137 and CD69, which associated with T cell activation, were upregulated significantly in CAR positive-subpopulation, while these molecules basically remained unchanged in CAR negative-subpopulation comparing to untreated group, suggesting that CAR-T cells was stimulated by the corresponding antigens modified via membrane fusion, and the F-AgNPs cooperate with CAR-T cells in an antigen specific way (Fig. 3d). The cooperative cytotoxicity assay indicated that the F-AgNPs mediated antigen modification effectively increased the susceptibility of target cells towards corresponding CAR-T cell cytoxicity in a dose dependent manner. Meanwhile, T cells without anti-EvIII CAR transfection were set as control. Cytotoxicity of untransfected T cells basically remained unchanged towards tumor cells treated with F-AgNPs of different concentrations, which verified again that F-AgNPs cooperate with CAR-T cells in an antigen-specific way, which dependent on recognition and engagement between modified antigens and corresponding CAR constructs (Fig. 3e and Additional file 1: Fig. S6b–d). To better simulate in vivo tumor tissue, HGC27 MCTs were constructed for cytotoxicity assay. The cell viability of tumor spheroid was quantified by visualizing tumor cell killing. Representative images indicated that neither F-AgNPs nor CAR-T cells alone exerted significant cytotoxicity towards MCTs without corresponding targets. In contrast, the cooperation of F-AgNPs and CAR-T cells increased dead cells and decreased living cells throughout the spheroids (Fig. 3f). Above ex vivo studies initially confirmed our hypothesis that F-AgNPs mediated antigen modification could efficiently activate corresponding CAR-T cells and cooperate to exert antitumor response.

### Biodistribution and tumor targeting of F-AgNPs in vivo

**Fig. 4:**
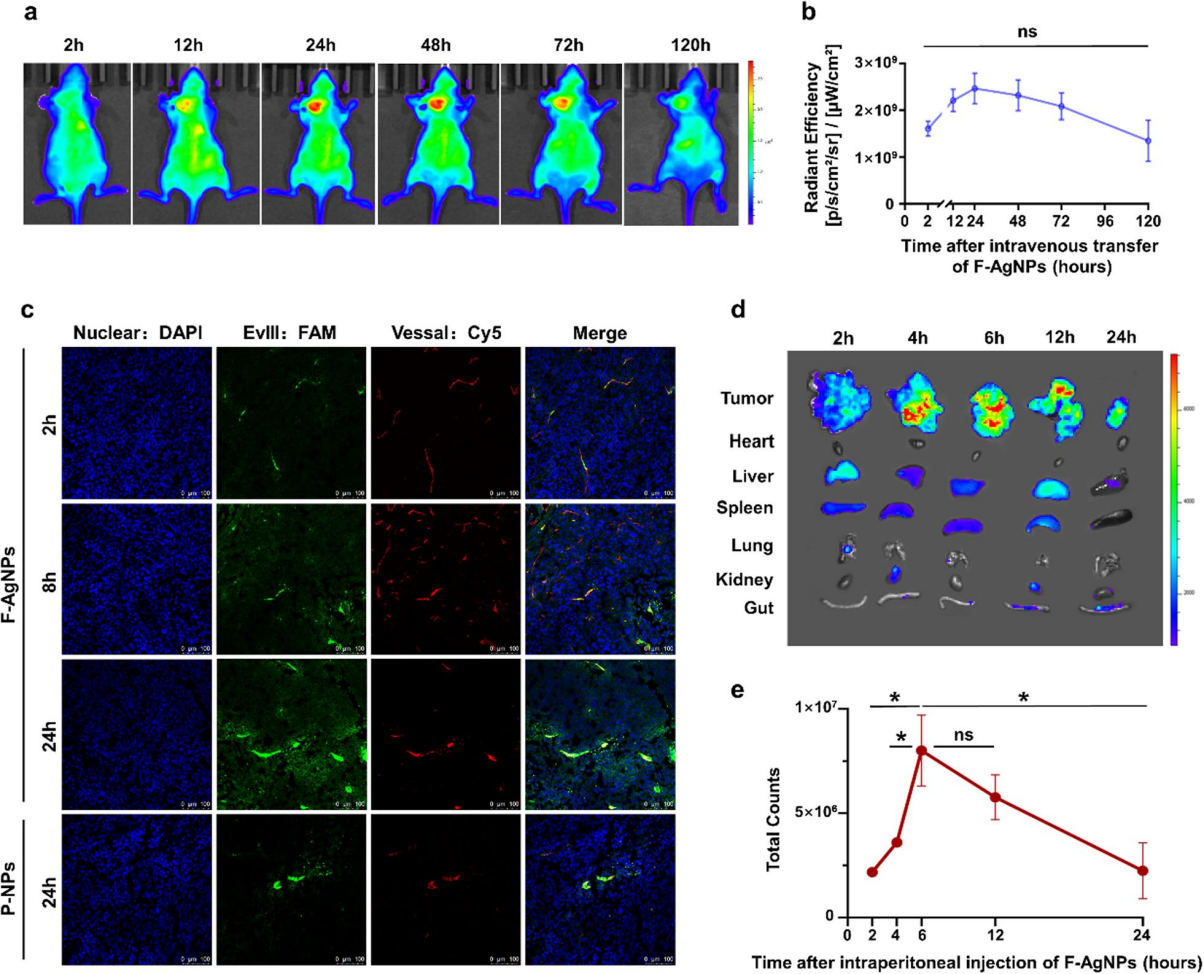
Biodistribution and tumor targeting of F-AgNPs in vivo. **a**, **b** The dynamics of DiR signals over time within tumor tissue after systemically delivering DiR-labeled F-AgNPs. Data are represented as mean ± s.e.m. *n* = 3. Student’s *t* test was used for statistical analysis. ns not significant. **c** Evaluation of in vivo antigen modification effects through observing the fluorescence images of MKN45 subcutaneous tumors sections after intravenous injection of F-AgNPs or P-NPs (FAM-labeled). Tumors were collected 2 h, 8 h and 24 h after delivery. Nucleus (DAPI, blue); antigen peptide (FAM, green); vessal (Cy5, red). Scale bar, 100 μm. **d**, **e** The dynamics of DiR signals over time in tumor and organs after intraperitoneal injection of DiR-labeled F-AgNPs. Data are represented as mean ± s.e.m. *n* = 4. Student’s *t* test was used for statistical analysis. ns not significant; *p < 0.05

The in vivo metabolism and biodistribution of F-AgNPs are crucial to evaluate the feasibility and safety of the cooperative remedy and explore an optimal schedule. MKN45 subcutaneous tumor model was established, and DiR was applied to track the in vivo dynamic distribution of systemically delivered F-AgNPs. Whole-body near infrared imaging demonstrated that F-AgNPs accumulated increasingly at tumor site after intravenous administration, and reached the peak in 24 h after transferring. Then F-AgNPs in tumor tissue metabolized gradually and return to original level about 120 h after administration (Fig. 4a, b). Consistent with the in vivo dynamics observed, antigens modified on in vivo tumor tissue increased gradually within 24 h after transferring, and the antigen modification efficiency of F-AgNPs was far better than conventional P-NPs in both intensity and range. (Fig. 4c). Tumors and organs were collected 24 h after systemic delivery, when the accumulation of F-AgNPs reached its peak in tumor lesions. The quantification of DiR signal revealed that, besides tumor lesion, F-AgNPs also aggregated in liver and spleen, in which reticuloendothelial system were of great abundance (Additional file 1: Fig S7a). However, distinct from antigen peptides mainly modified on cell membrane in tumor tissue, fusogenic nanoparticles and their loading antigens accumulated in mononuclear phagocytic system of liver tend to be rapidly taken up into cytoplasma by phagocytosis, minimizing the exposure onto the cell membrane (Additional file 1: Fig S7b,c) [15, 16]. In disseminated peritoneal tumor model injected intraperitoneally with DiR-labeled F-AgNPs, the accumulation of F-AgNPs in tumor nodes reached its peak 6 h after administration (Fig. 3d, e). The strongest accumulation was observed in tumor nodes at all time point, while DiR signals could hardly be detected in other organs expect for mild signals observed in liver and spleen (Additional file 1: Fig S8). Above results suggested that F-AgNPs mediated in vivo antigen modification in a relatively tumor membrane specific manner, providing the feasibility to cooperate with CAR-T cells via different *route*.

### F-AgNPs mediated in situ antigen modification cooperates with CAR-T cells adoptive transfer, the TRUE CAR-T cell therapy in solid tumors

**Fig. 5:**
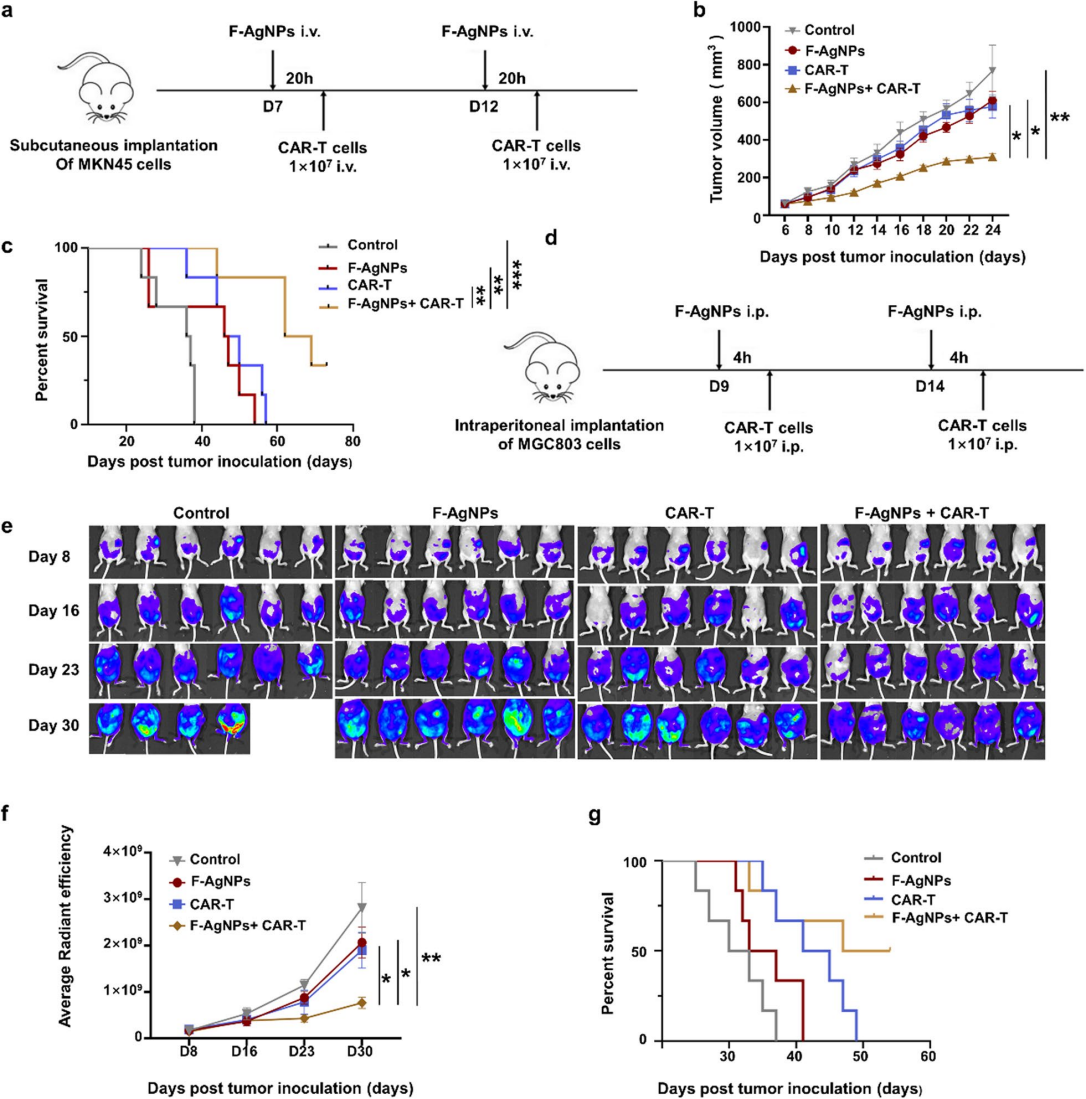
F-AgNPs mediated antigen modification cooperates with CAR-T cell therapy in vivo. **a** Schematic illustration of treatment process in subcutaneous tumor model. **b**, **c** Enhanced antitumor effect of F-AgNPs mediated antigen modification cooperative with CAR-T cell therapy in a subcutaneous MKN45 tumor model. Tumor growth profiles (**b**) and survival curve (**c**) of mice treated with PBS, F-AgNPs, EvIII CAR-T, F-AgNPs + EvIII CAR-T respectively. Tumor volume were analyzed with one-way ANOVA. Data are represented as mean ± s.e.m. *n* = 6. Survival curves were analyzed with log-rank test. *n* = 6. *p < 0.05; **p < 0.01; ***p < 0.001. **d** Schematic illustration of treatment process in peritoneal metastasis tumor model. **e–g** Enhanced antitumor effect of F-AgNPs mediated antigen modification cooperative with CAR-T cell therapy in a disseminated peritoneal MGC803 tumor model. Tumor growth profiles (**e**, **f**) and survival curve (**g**) of mice treated with PBS, F-AgNPs, EvIII CAR-T, F-AgNP s + EvIII CAR-T respectively. Data are represented as mean ± s.e.m.., *n* = 6. A one-way ANOVA was used for statistical analysis. *p < 0.05; **p < 0.01; ***p < 0.001

With the cellular membrane antigen modification and in vivo tumor targeting capabilities established, we next evaluated the therapeutic efficacy of the cooperative remedy combing F-AgNPs with CAR-T cells adoptive transfer. The in vivo antitumor effect was first studied in mice bearing MKN45 xenograft tumor model (Fig. 5a). Neither F-AgNPs alone nor CAR-T cells without corresponding targets exerted significant suppression on tumor growth compared to untreated group. As expected, CAR-T cell therapy cooperated with F-AgNPs elicited maximum antitumor efficacy in both tumor growth control and prolonging survival, without apparent side effects being observed (Fig. 5b, c, and Additional file 1: Fig. S9a–c). Peritoneal metastases are commonly observed in several malignancy and generally lead to poor prognosis and lower median survival. To better simulate clinical practice, disseminated peritoneal tumor model was established using MGC803 transfected with luciferase activity (Fig. 5d). Dynamic bioluminescence imaging observation demonstrated that none of the studied formulations exhibited significant tumor control relative to the untreated group within a week after treatment. Observation in two weeks showed that only the combination treatment group effectively inhibited tumor growth, and the disparity of tumor growth between experimental groups expanded gradually (Fig. 5e, f, Additional file 1: Fig. S10). At the end point of observation, 33% (2/6) mouse died in untreated group, while cooperation of F-AgNPs and CAR-T cells exhibited superior tumor control compared to either single treatment group and effectively prolonged survival (Fig. 5e–g). No obvious toxicity or alterations in body weight has been observed in these experimental groups (Additional file 1: Fig. S11a, b). The above results indicated that F-AgNPs mediated antigen modification successfully provided targets for CAR-T cells, and functioned to mediate the recognition, engagement and finally antitumor efficacy of CAR-T cells towards tumors without inherent corresponding targets, with favorable safety, feasibility and efficiency.

## Discussion

Here in our study, the concept of universal CAR-T cell therapy was creatively realized through tumor cell target-redirecting mediated by exogenous antigen modification, instead of reforming CAR-T cell itself. The fusogenic in situ antigen modification nano-platform played a crucial role in this therapeutic strategy. The antigen modification pattern exhibited a certain tumor membrane selectivity, which was consistent with previous studies[24]. Such tumor specificity was possibly attributed to the change of cell membrane during malignant transformation. Tumor cell membrane is more negatively charged, less ordered and held better liquidity than normal cells, thus may better interact with F-AgNPs comprising cationic lipid DOTAP and increasing the probability of fusion [17, 25, 26] (Additional file 1: Fig. S12a–e). Moreover, extracellular vesicles (EVs) are known to mediate intercellular lipid transfer, which enabled loaded antigens to spread throughout tumor tissues [27–29]. The tumor cell-derived EVs interacted more efficiently with tumor cells compared with other types of cells, thus indicating preferential antigen modification of tumor cells in tumor microenvironment [24, 30]. Furthermore, by introducing tumor environment-responsive elements, such as gelatinase cleavable peptide which responds to tumor microenvironment due to multiple enzymes overexpressed, the tumor-specificity of nanoparticles might be further optimized [31, 32]. It is worth noting that the antigen modification strategy is not restricted to nanosystems. For instance, pH low insertion peptide which can selectively assemble onto the membrane of tumor cells by responding to the acidic tumor microenvironment also serve as a potential strategy for tumor membrane specific antigen modification[33]. With the specific tumor cell membrane in situ antigen modification, CAR-T cells can be redirected towards tumors originally without corresponding targets and functioned universally, completely getting rid of the restriction of inherent tumor antigen profile, thus holding an even wider applicable population. In addition, F-AgNPs mediated exogenous antigen modification exhibited favorable flexibility. The ‘expression’ level of exogenous antigens was controllable through dosage adjustment, which may help to improve the unsatisfactory therapeutic efficacy of CAR-T cells resulted from insufficient expression of target antigens. Repetitive administration of F-AgNPs is also expected to circumvent the loss of target antigen under therapeutic pressure, thus avoiding acquired resistance towards CAR-T cell therapy.

Considering the diversity of biological delivery systems and immunotherapeutic strategies, the cooperative strategy is not restricted to nanosystems and CAR-T cells. In a broader context, our work focused on the paucity and heterogeneity of targetable tumor antigens, a common dilemma faced by multiple treatment modalities of solid tumors, and provided a tumor cell membrane-selective antigen modification platform for further development of inherent antigen-independent antitumor remedies. The in situ tumor membrane modification strategy can be further extended to various immune effector cells, including innate or acquired immune cells. By integrating different immune ligands, it was expected to demonstrate the potential as a general platform for the design of novel immunotherapies [34]. Above all, the establish of TRUE CAR-T cell therapy and the cooperative strategy provide new perspectives for the future development of immunotherapy for solid tumors.

## Conclusion

In summary, based on fusogenic nanosystem, we developed a TRUE CAR-T cell therapeutic strategy which can redirect CAR-T cells towards tumors without corresponding targets, and exert antitumor effects while get rid of the restriction of inherent tumor antigen profile. By incorporated into fusogenic nanoparticles, antigen peptides were efficiently modified onto tumor cell membrane by direct plasma membrane fusion, with a certain tumor membrane selectivity and better tumor penetration. F-AgNPs mediated in situ antigen modification successfully redirected CAR-T cells toward tumor cells without inherent corresponding antigens, motivated T cell response and cytolytic activity in an antigen specific manner. The F-AgNPs mediated in vivo antigen modification exhibited relatively tumor selectivity via both intravenous and intraperitoneal administration, and the cooperation of F-AgNPs and CAR-T cells demonstrated significant suppression on tumor growth and prolonged survival in different tumor models without obvious toxicity. These data presented provide a proof-of-principle for the TRUE CAR-T cell therapy with application advantages and translational potential in immunotherapies for solid tumors.

## Supplementary Information

**Additional file 1.** Supplementary figures.

## Data Availability

The datasets used and/or analyzed during the current study are available from the corresponding author on reasonable request.

## Abbreviations

TRUE CAR-T: Target-redirected universal chimeric antigen receptor T
CAR: Chimeric antigen receptor
F-AgNPs: Fusogenic antigen loaded nanoparticles
DMPC: 1,2-Dimyristoyl-sn-glycero-3-phosphocholine
DOTAP: 1,2-Dioleoyl-3-trimethylammonium-propane
DSPE-PEG-Mal: 1,2-Distearoyl-sn-Glycero-3-Phosphoethanolamine-N-[Maleimide (polyethylene-Glycol)-3400]
HUVECs: Human umbilical vein endothelial cells
MALDI-TOF MS: Matrix-assisted laser desorption/ionization-time-of-flight-mass spectrometry
FACS: Flow cytometry staining
PBMCs: Peripheral blood mononuclear cells
EGFR vIII: Epidermal growth factor receptor variant III
DLS: Dynamic light scattering
MCTs: Multicellular tumor spheroids
NF-NPs: Cationic nonfusogenic nanoparticles
P-NPs: PEGylated nonfusogenic nanoparticles
EVs: Extracellular vesicles
