# Supplementary material for: In situ antigen modification-based target-redirected universal chimeric antigen receptor T (TRUE CAR-T) cell therapy in solid tumors
Source: J Hematol Oncol. 2022 Mar 18;15:29. doi: 10.1186/s13045-022-01246-y (PMC8932190; doi:10.1186/s13045-022-01246-y)
Supplement: Supplementary file 1 — Additional file 1. Supplementary figures. [file 13045_2022_1246_MOESM1_ESM.docx]

**
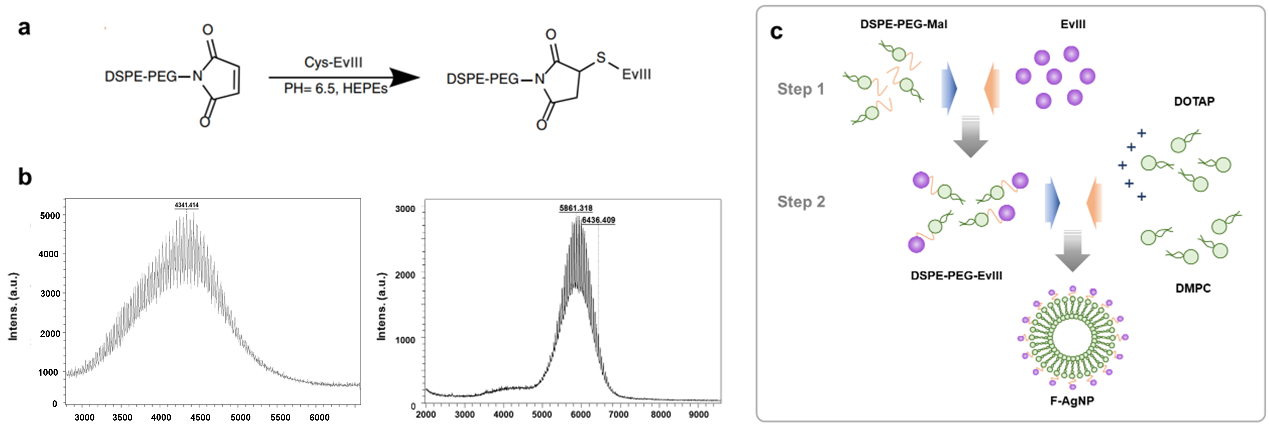
**

**Supplementary Fig. 1 | Synthesis of DSPE-PEG-EvIII and** construction of F-AgNPs. a Synthesis of lipid-conjugated antigen peptides, DSPE-PEG-EvIII through Michael addition reaction. **b** The molecular weight of DSPE-PEG-Mal and reaction product, which indicates the successful connection of EvIII and DSPE-PEG-Mal. **c** F-AgNPs were constructed using the film hydration method.

**
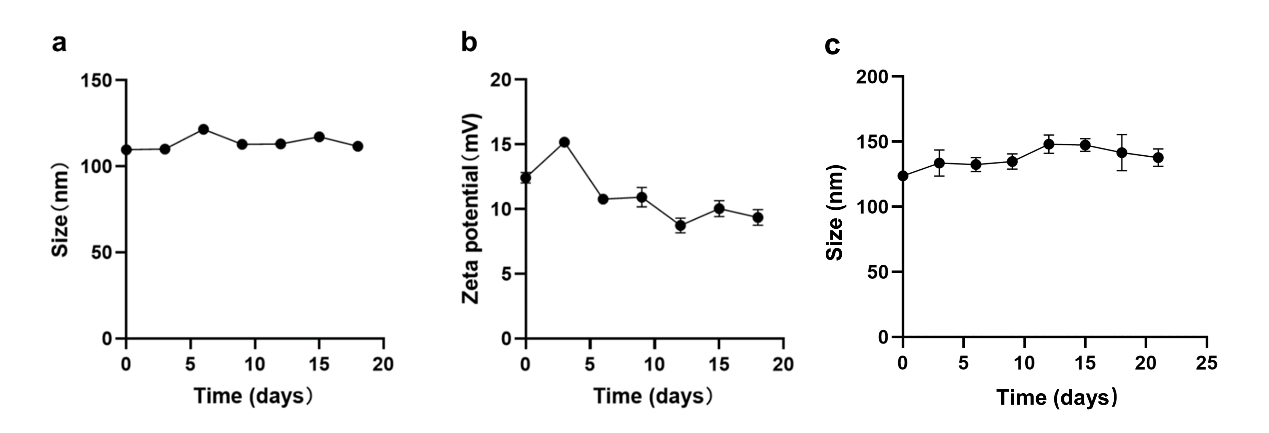
**

**Supplementary Fig. 2 | Stability of F-AgNPs.** **a,b** F-AgNPs could remain stable size (**a**) and surface charge (**b**) in phosphate buffer solution for up to 21 days at 4 °C. **c** F-AgNPs could remain stable size in blood plasma for up to 21 days at 4 °C. Data are represented as mean ± s.e.m.; n = 3.


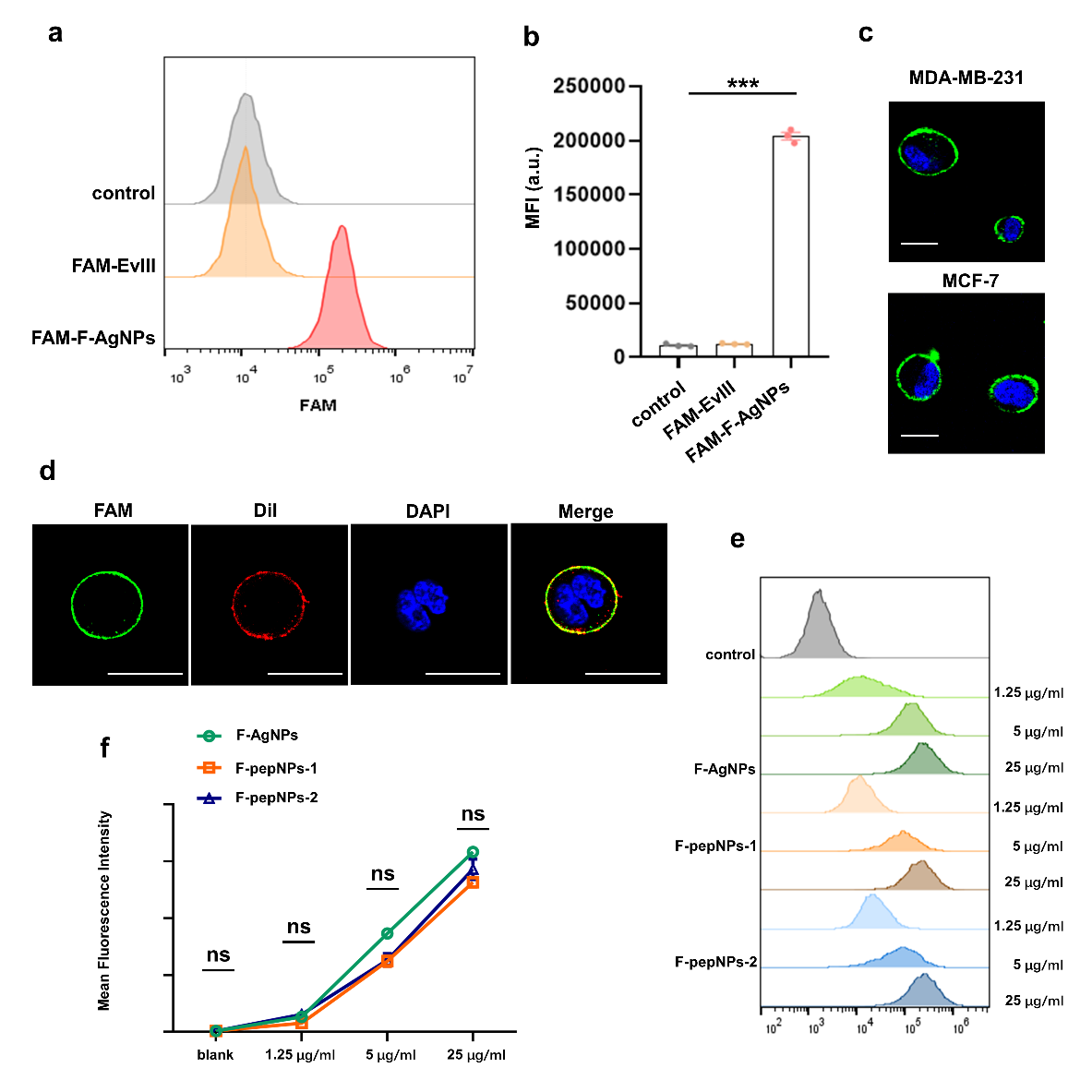


**Supplementary Fig. 3 | Fusogenic nanoparticles mediated modification with different peptides and F-AgNPs mediated modification of different cancer cells. a, b** Flow cytometry histograms (**a**) and quantitative analysis (**b**) of MDA-MB-231 without treatment (grey), incubated with FAM-EvIII (orange) or F-AgNPs (red). Data are presented as mean ± s.e.m., n = 3. Student’s t-test was used for statistical analysis. ***p < 0.001. **c** Confocal fluorescence images of F-AgNPs mediated antigen modification of MDA-MB-231 and MCF-7. Scale bar represents 20 μm. **d** Localization of antigen peptides modified by F-AgNPs on MDA-MB-468. Nucleus: DAPI (blue); antigen peptide: FAM (green); plasma membrane: DiI (red). Scale bar represents 20 μm. **e** Histograms of tumor treated with F-AgNPs, F-pepNP-1 (FAM-NYSKPTDRQYHF) and F-pepNP-2 (FAM-GNWDYNSQRAQLYNQ) of different dosage. **f** Quantitative analysis of modification efficacy of fusogenic nanoparticles loading peptides of different sequences. Data are represented as mean ± s.e.m.; n = 3. Student's t test was used for statistical analysis. ns not siginificant.


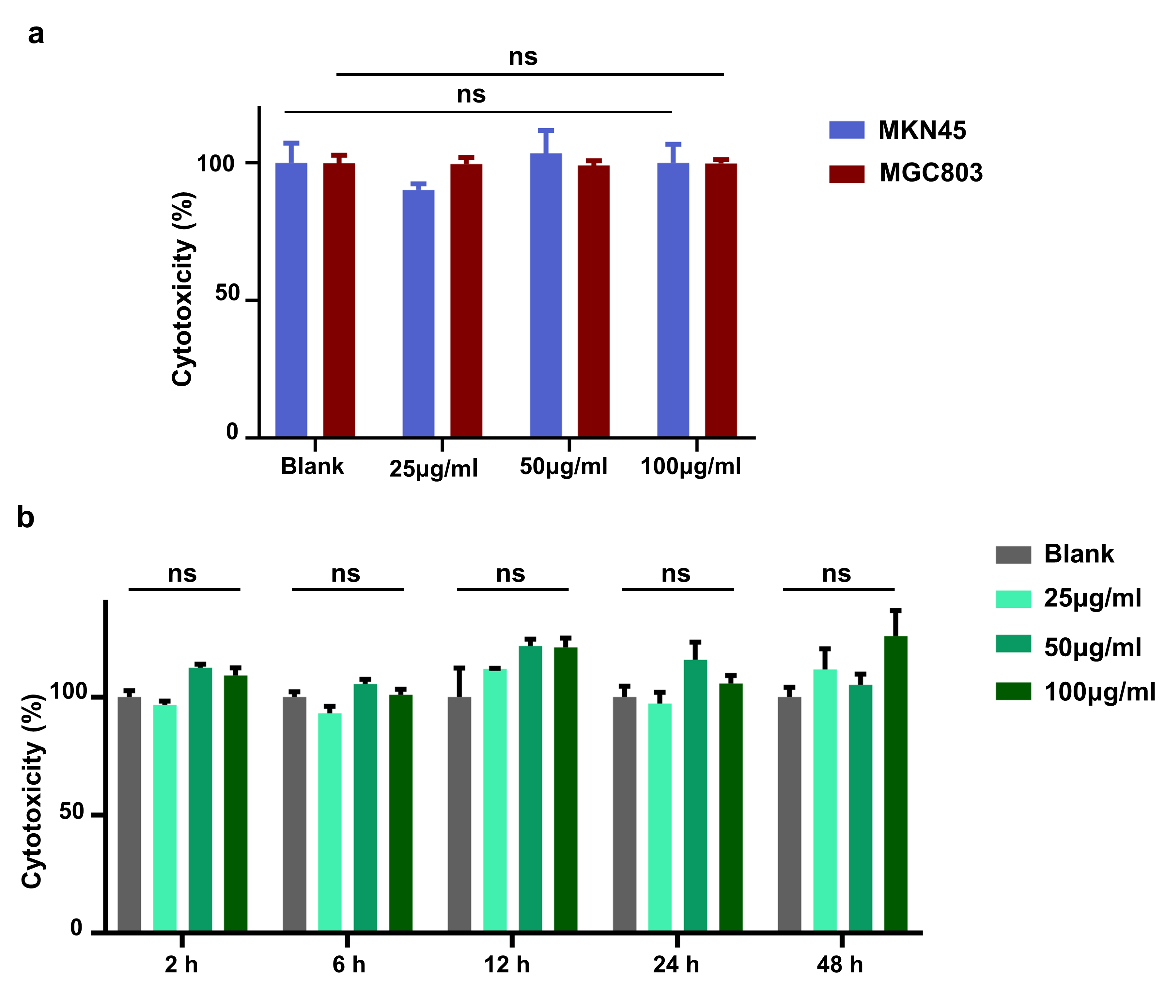


**Supplementary Fig. 4 | Biosafety of F-AgNPs mediated cell membrane modification. a** Viability of MKN45 and MGC803 gastric cancer cells after 45 min incubation with F-AgNPs in concentration far higher than working concentration (25 µg/ml) of 50 µg/ml and 100 µg/ml. Data are represented as mean ± s.e.m., n = 5. A one-way ANOVA was used for statistical analysis. ns, not significant. **b** Viability of MKN45 gastric cancer cells after 2, 6, 12, 24, 48 h incubation with F-AgNPs of 25, 50 and 100 µg/ml. Data represent mean ± s.e.m., n = 5. A one-way ANOVA was used for statistical analysis. ns, not significant.


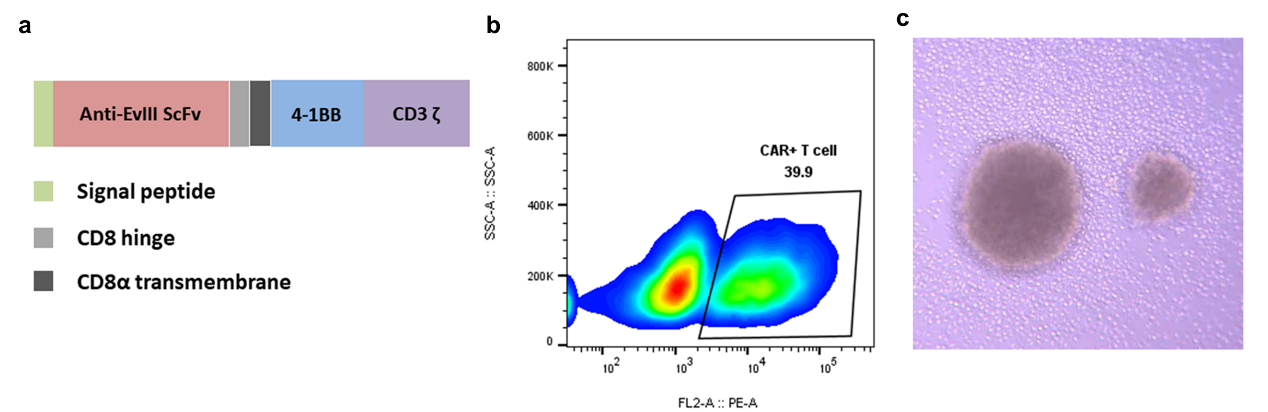


**Supplementary Fig. 5 | Construction and transfection of EvIII CAR-T cells.** **a** Schematic illustration of EvIII CAR construct. **b** Transfection efficiency of EvIII CAR-T cells 7 days after electrotransfection. **c** Morphology observation 7 days after electrotransfection indicated that EvIII CAR-T cells were in good condition.


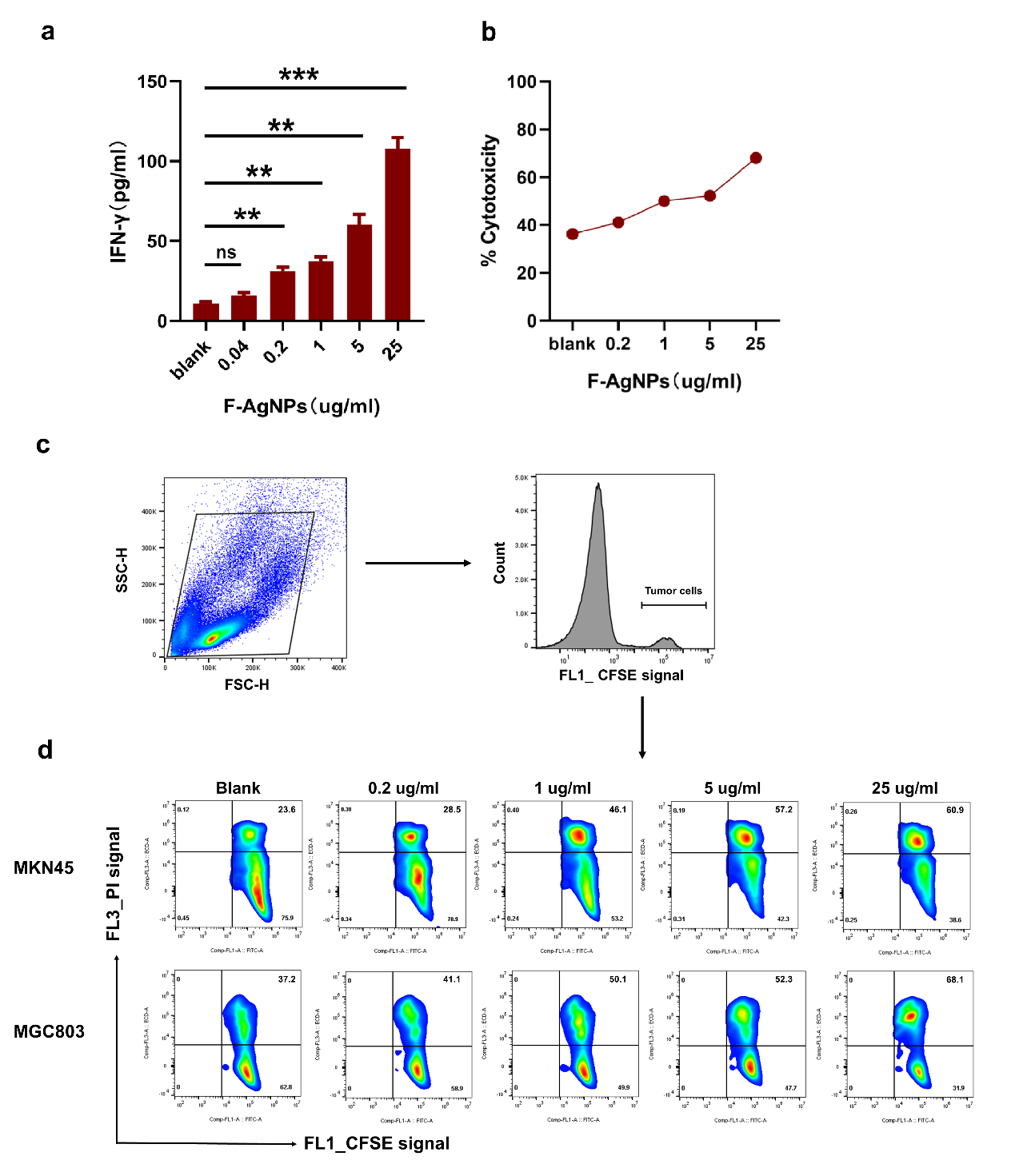


**Supplementary Fig. 6 | Evaluation of *in vitro* cooperative antitumor response of TRUE-CAR-T therapeutic strategy. a** IFN-γ secretion of EvIII CAR-T cells after 24 h incubation with tumor cells (MGC803) treated by F-AgNPs of various concentration. Data represent mean ± s.e.m., n = 3. Student’s t-test was used for statistical analysis. ns not significant; **p < 0.01; ***p < 0.001. **b** Evaluating cytotoxicity of EvIII CAR-T cells towards F-AgNPs treated tumor cells (MGC803) and exploring the dose-effect relationships. E:T=10:1 **c** The gating methods for flow cytometry analysis of CFSE/PI assays. **d** Representative flow cytometry plot of CFSE/PI assays evaluating cytotoxicity of TRUE- CAR-T cell therapy towards tumor cell (MKN45 and MGC803).


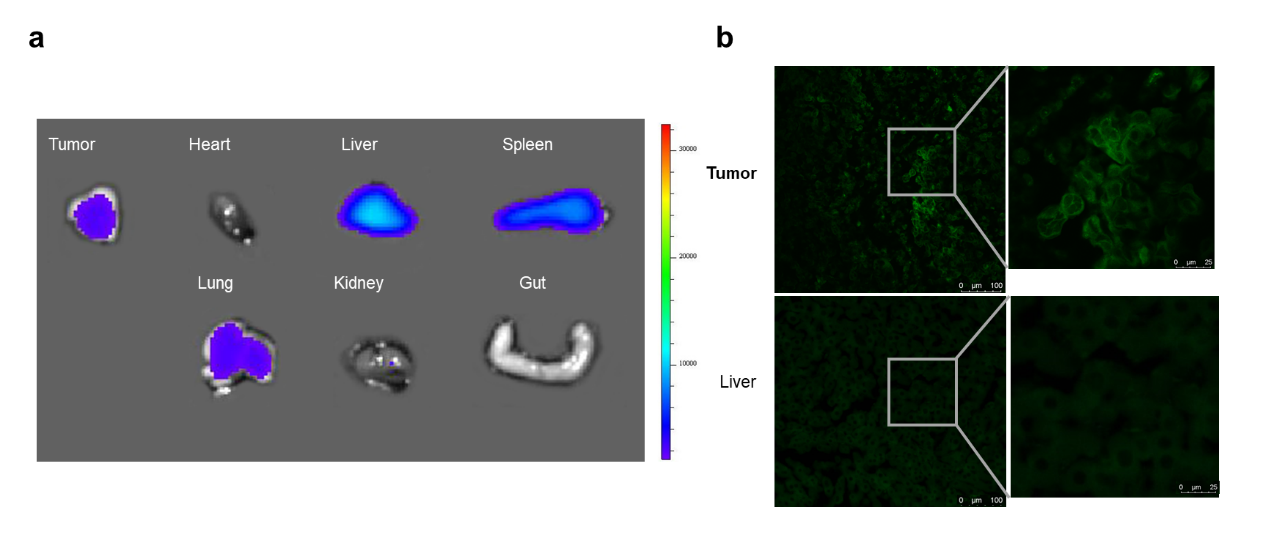


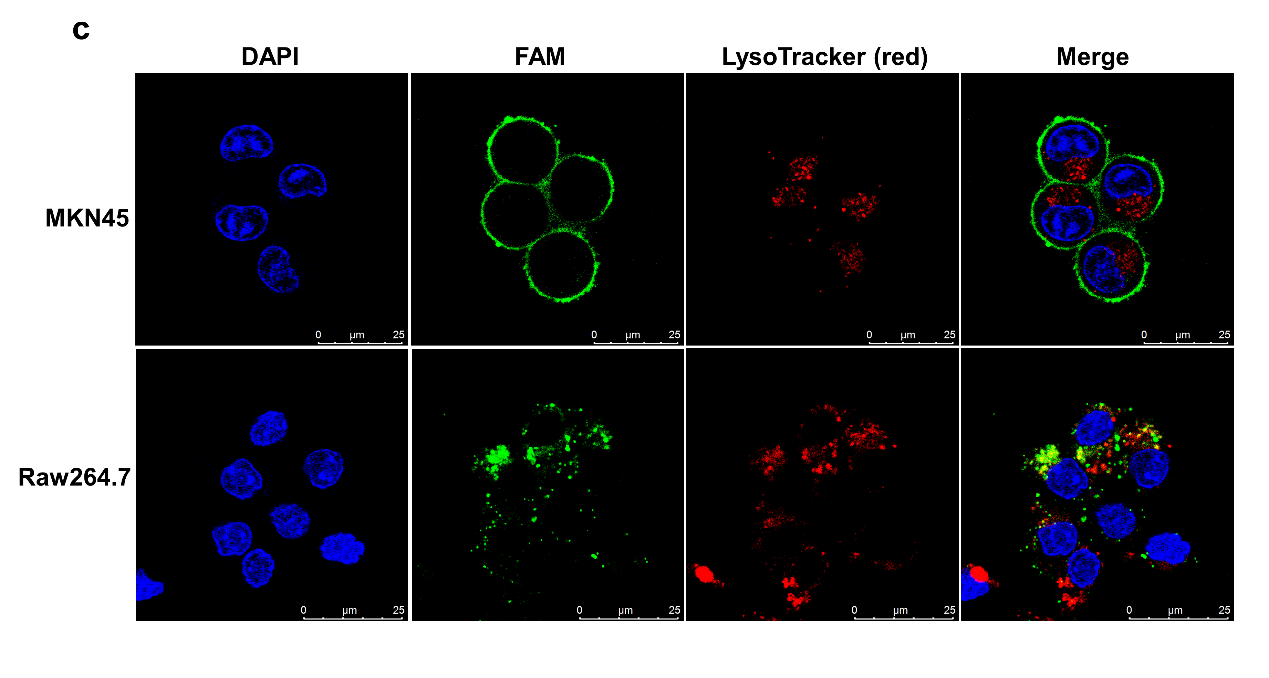


**Supplementary Fig. 7 | F-AgNPs accumulation and modification in subcutaneous tumor and organs after intravenous administration and the different interaction pattern of F-AgNPs in tumor cells and phagocytes.** **a** Near infrared imaging of DiR signals accumulation in tumor and other organs 24h after intravenous transfer of F-AgNPs. **b** The location and modification pattern of FAM-antigen peptides was compared between tumor tissue and liver. **c** Confocal fluorescent microscopic images of tumor cells (MKN45) and macrophages (Raw264.7) after treated with FAM-F-AgNPs for 45 min. Nucleus: DAPI (blue); antigen peptide: FAM (green); lysosome: LysoTracker (red). Scale bar represents 25 μm.


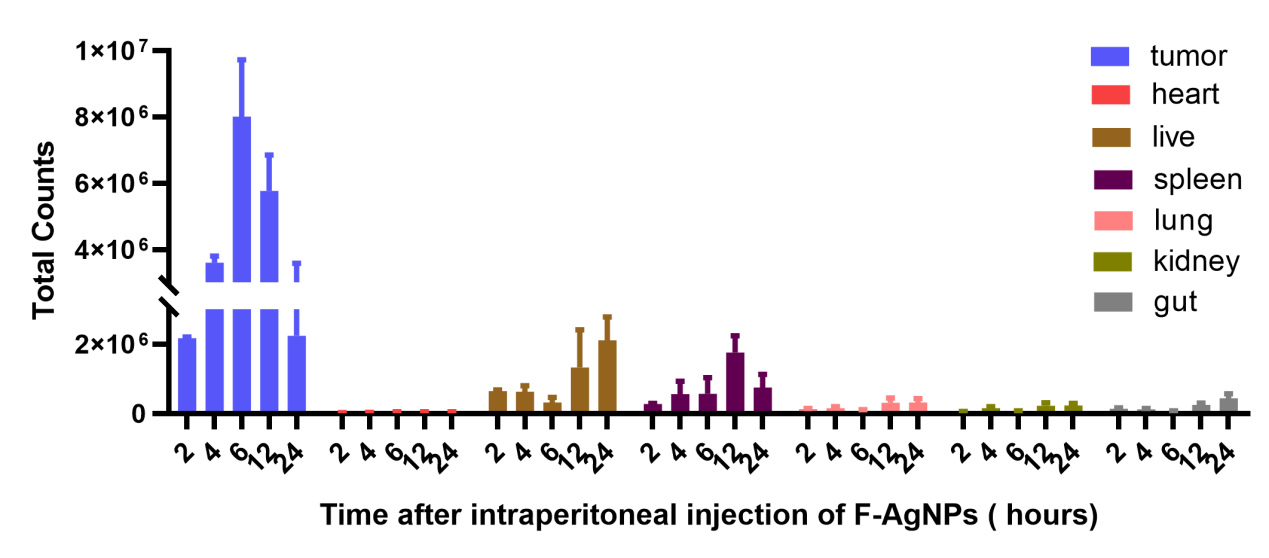


**Supplementary Fig. 8 | *In vivo* biodistribution of F-AgNPs in disseminated peritoneal tumor model.** Quantification of DiR signals in peritoneal metastases and various organs at different time points after F-AgNPs intraperitoneal injection. Data are represented as mean ± s.e.m., n = 4.

.


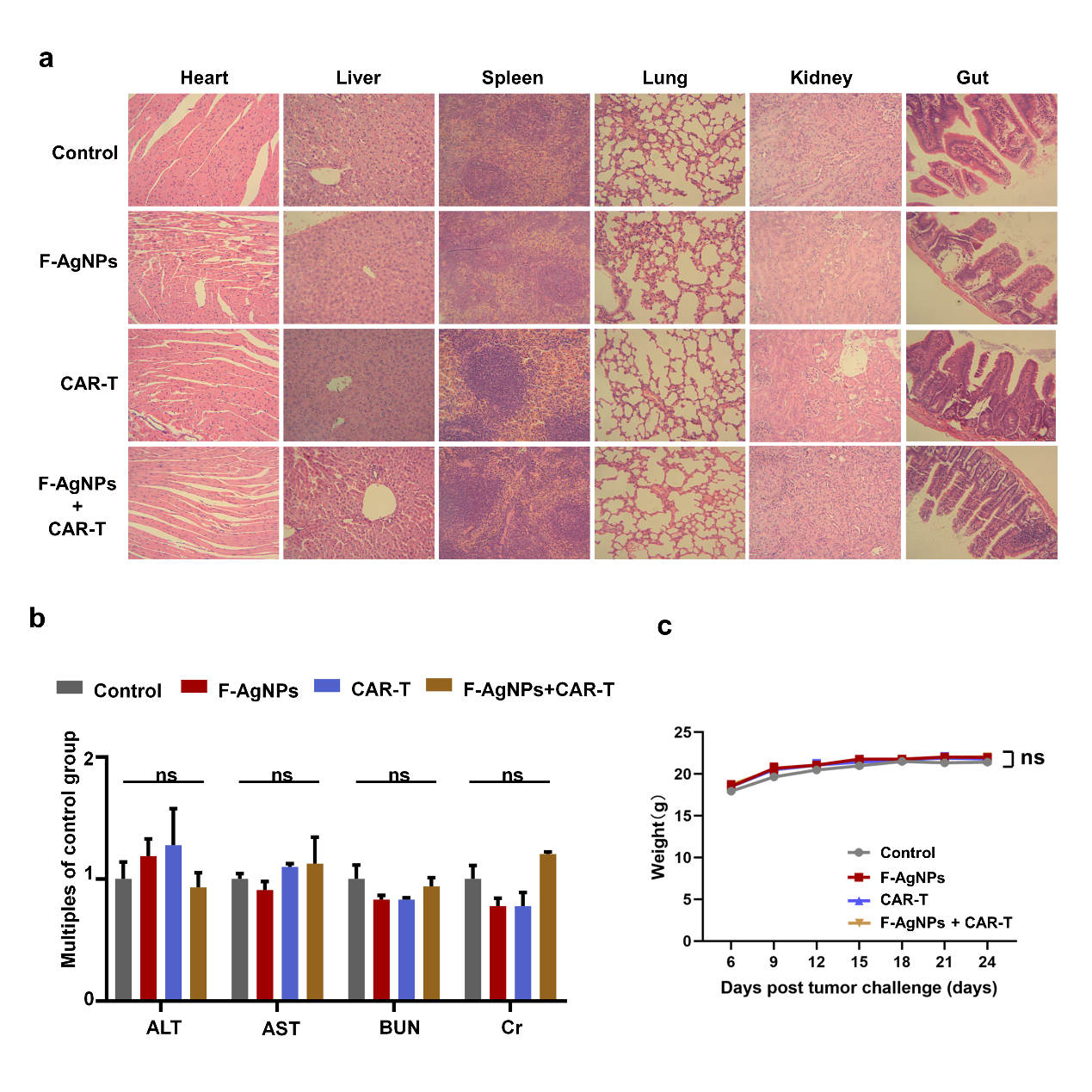


**Supplementary Fig. 9|** **Safety assessment of the cooperative therapy of F-AgNPs and CAR-T cells through intravenous administration.** **a** H&E staining of major organs of the representative mouse in every treatment group, indicating no severe toxicity of vital organs was observed. **b** Assessment of liver and kidney function at two weeks post start of treatment. Data are represented as mean ± s.e.m.; n = 3. Student's t test was used for statistical analysis. ns not significant. AST, Aspartate aminotransferase; ALT, Alanine aminotransferase; BUN, Blood urea nitrogen; Cr, Creatinine. **c** Weight records of mice in every treatment group, no obvious weight loss was observed. Data are represented as mean ± s.e.m.; n = 6. Student's t test was used for statistical analysis. ns not significant.


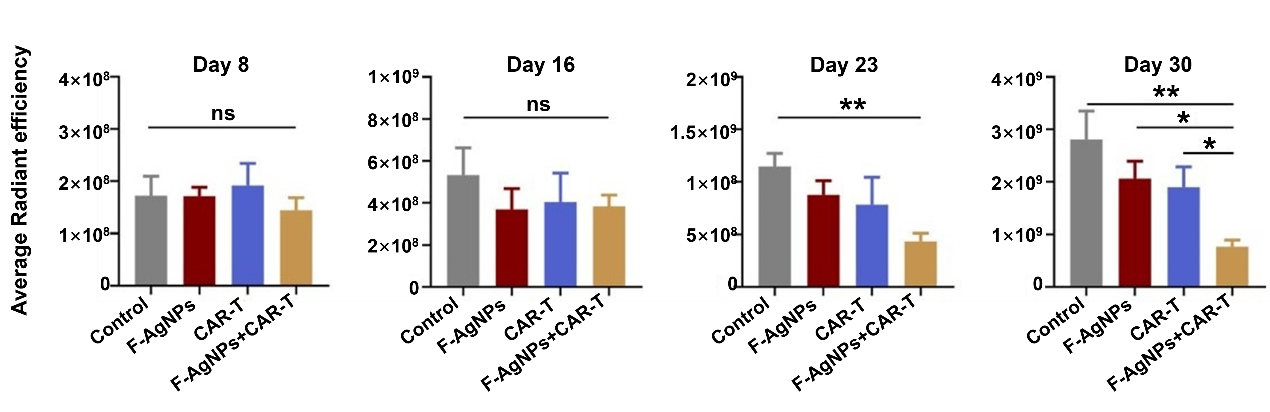


**Supplementary Fig. 10|** **Enhanced tumor suppression effect of TRUE CAR-T cell therapy in a disseminated peritoneal MGC803 tumor model**. Tumor growth profiles were evaluated in Day 8, 16, 23, 30 respectively for mice treated with PBS, F-AgNPs, EvIII CAR-T, F-AgNP s+ EvIII CAR-T respectively. Data are represented as mean ± s.e.m.., n = 6. A one-way ANOVA was used for statistical analysis. ns not significant; *p < 0.05; **p < 0.01; ***p < 0.001.

**
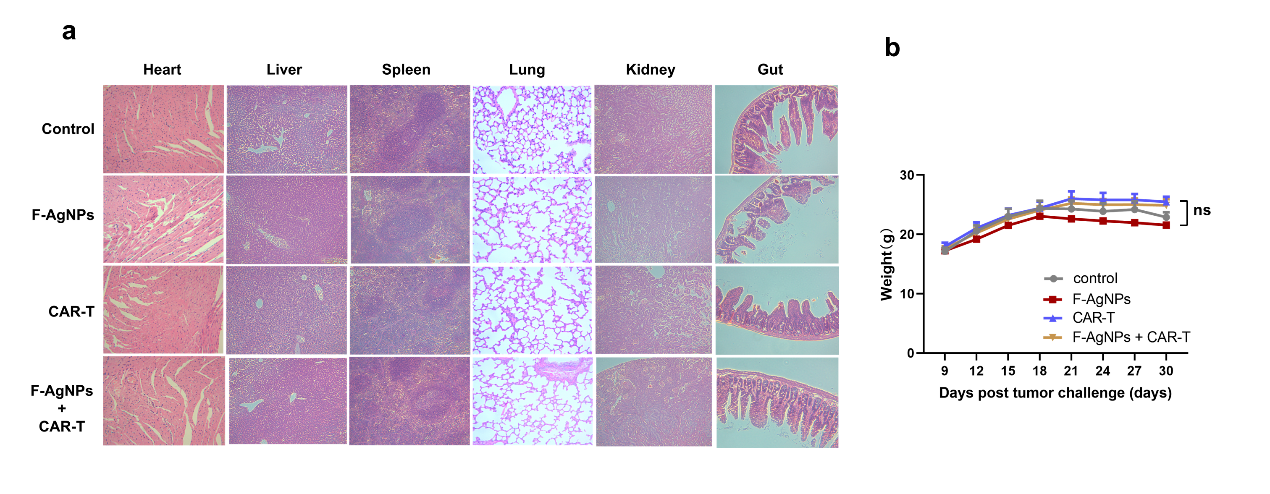
**

**Supplementary Fig. 11** **| Safety assessment of the cooperative therapy of F-AgNPs and CAR-T cells through** **intraperitoneal administration. a** H&E staining of major organs of the representative mouse in every treatment group, indicating no severe toxicity of vital organs was observed. **b** Weight records of mice in every treatment group, no obvious weight loss was observed. Data represent mean ± s.e.m., n = 6. Student's t test was used for statistical analysis. ns not significant.


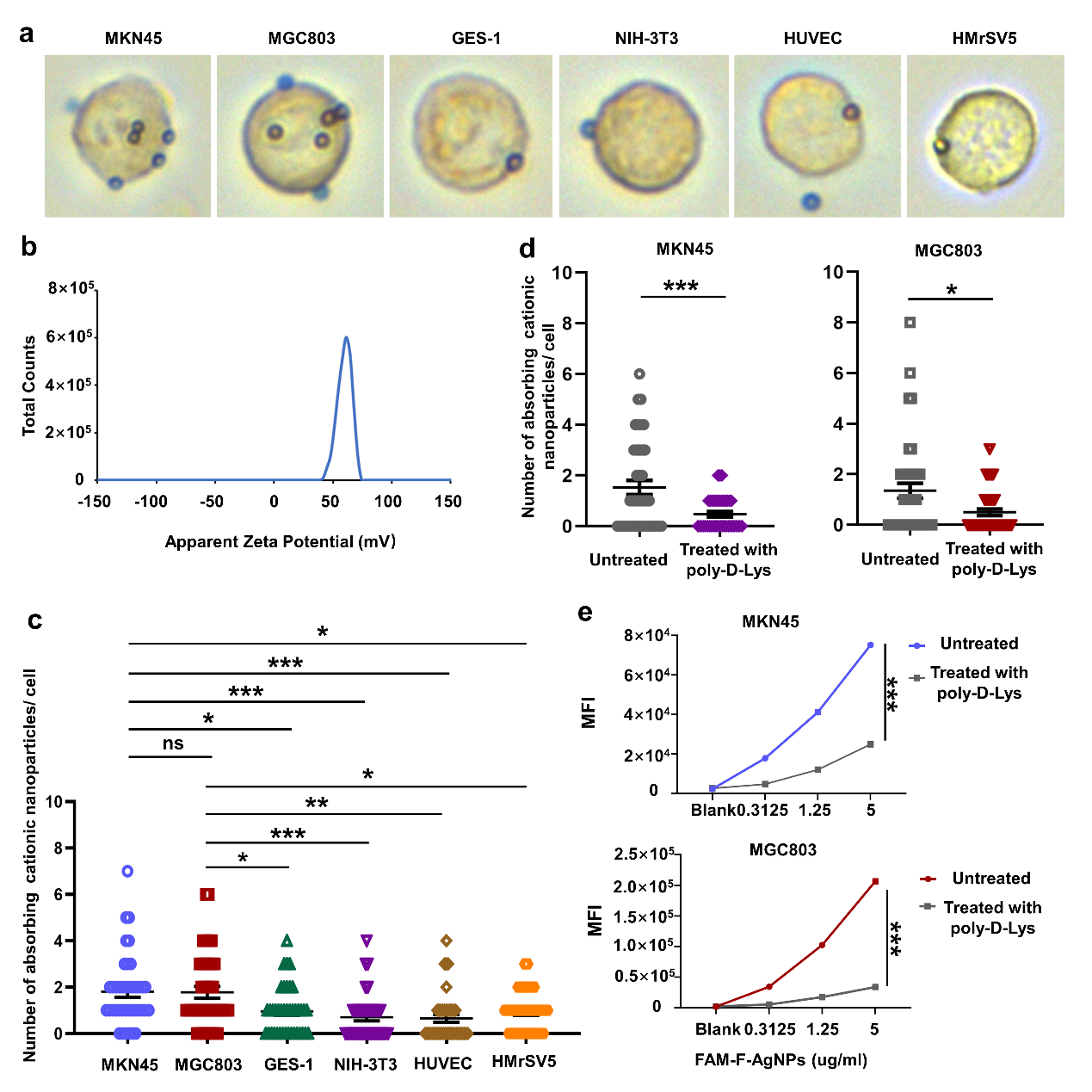


**Supplementary Fig. 12 |** **Mechanism analysis of tumor cell membrane-selectivity of F-AgNPs mediated modification.** **a** Representative microscope images reflecting cationic nanoparticles adsorption of multiple cells. **b** Zeta potential of commercial cationic nanoparticles. **c** Quantitative analysis of cationic nanoparticles adsorption on multiple cells. Data are represented as mean ± s.e.m.; n = 40. A one-way ANOVA was used for statistical analysis. ns not significant; *p < 0.05; **p < 0.01; ***p < 0.001. **d** Quantitative analysis of cationic nanoparticles adsorption of tumor cells with or without poly-D-lysine treatment (blocking the positive charge of cell surface). Data are represented as mean ± s.e.m.; n = 40. Student's t test was used for statistical analysis. *p < 0.05; ***p < 0.001. **e** Quantitative analysis of F-AgNPs-mediated modification efficiency of tumor cells with or without poly-D-lysine treatment (blocking the positive charge of cell surface). Data are represented as mean ± s.e.m.; n = 3 Student's t test was used for statistical analysis. ***p < 0.001.
